# Oil Palm Shell-Derived Activated Carbon: Adsorption Kinetics, Thermodynamics, and Interaction Mechanism for Lufenuron 50-EC Pesticide

**DOI:** 10.1021/acsomega.4c10096

**Published:** 2026-01-27

**Authors:** David Nuñez-Vargas, Juan Barraza-Burgos, Juan Guerrero-Perez, Luis Diaz, Ajay K. Dalai, Venu Babu Borugadda

**Affiliations:** † 28006 Universidad del Valle, Chemical Engineering School, Ciudad Universitaria Meléndez, Calle 13 # 100-00, Cali, A.A. 25360, Columbia; ‡ 125412 Fundación Universitaria del Área Andina, Valledupar Research and Development Center (CIDVA), Transversal 22 Bis #4-105, Valledupar, Cesar 200001, Colombia; § 7235 University of Saskatchewan, College of Engineering, 57 Campus Drive, Saskatoon, Saskatchewan S7N 5A2, Canada

## Abstract

Activated carbon (AC) was synthesized from oil palm shell (OPS) through physical (AC-800-2 and AC-900-2) and chemical (AC-750-1.5-3:1 and AC-800-1-2:1) activation processes in a carbon dioxide atmosphere. KOH was used as an activating agent in the impregnation process in the case of chemical activation. The synthesized ACs were characterized by proximate and ultimate analysis, Fourier transform infrared (FTIR) spectroscopy, X-ray diffraction (XRD), Raman spectroscopy, scanning electron microscopy (SEM), and textural properties of the samples. The tested properties, including surface area (*S*_BET_), pore volume (V_T_), and average pore size (S_P_), were determined by the Brunauer-Emmet-Teller (BET) method and the Barrett-Joyner-Halenda (BJH) model. Lufenuron adsorption results demonstrated that AC-900-2 achieved the highest lufenuron removal yield of 96.9%. The samples were best represented overall by the pseudo-second-order kinetic model, with *R* ^2^ values between 0.91 and 0.99. This suggests that the lufenuron adsorption process onto the activated carbons produced in this study was related to the chemisorption process. In addition, this adsorption process was spontaneous, exothermic, and exhibited a high probability of reversibility for samples AC-900-2, AC-750-1.5-3:1, and AC-800-1-2:1, with van der Waals forces and hydrogen bonds playing a significant role in the interaction between lufenuron and AC. In contrast, for sample AC-800-2, the adsorption process required an increase in temperature to become spontaneous, and the process was endothermic and irreversible. In general, the high percentages of adsorption observed in the AC produced by physical and chemical activation could be explained by the strong interactions of the surface functional groups with lufenuron. In the case of physically activated carbon, the high surface area provides more sites available for these interactions. In the case of chemically activated carbon , although the surface area is lower, the functional groups introduced using KOH improve the adsorption capacity.

## Graphical abstract

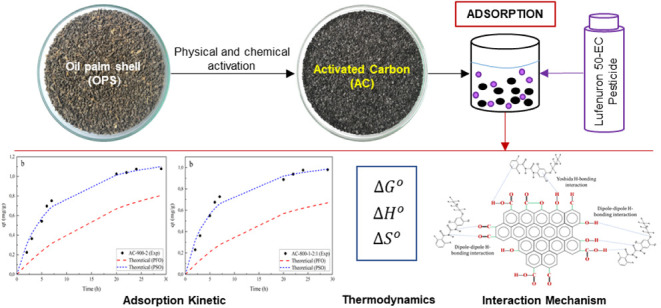

## 1 Introduction

The constant and excessive use of pesticides in agricultural crops has become a problem that grows as it continues to be the main control and prevention measure against the threat of production loss due to the appearance of undesirable pests. The accumulation, by deposition, of these pesticides in the environment threatens ecological integrity by disturbing water bodies, air, and soil, altering their physicochemical properties, pH, salinity, and alkalinity, resulting in soil infertility and endangering the survival of flora and fauna.^1,2^ It should be noted that long-term contact with pesticides generates serious health consequences; Parkinson’s, multiple sclerosis, aging, cancer, Alzheimer’s, diabetes, cardiovascular diseases, and chronic kidney diseases are some of the conditions associated with the pesticide–human relationship.^3,4^

Various adsorbents and methods have been used for the adsorption of pesticides: polymeric adsorbents,^5^ calcined hydrotalcite,^6^ organohydrotalcites,^7^ oil shale ash,^8^ watermelon peel,^9^ goethite and goethite coated with humic acid,^10^ biochar and charcoal untreated and treated with phosphoric acid,^11^ plasmonic nanoparticles,^12^ mushroom *Pleurotus mutilus*,^13^ functionalized magnetic nanoparticles,^14^ P-doped biochar,^15^ metal–organic frameworks,^16^ among others. Parallel to the aforementioned adsorbents, the adsorption of pesticides on activated carbons has been worked on^17–19^ derived from different organic precursors such as olive seed, corn cobs, rapeseed stalks and soybean stalks,^20^ banana stem,^21^ hemp fiber waste,^22^ and oil palm shell,^23^ demonstrating that the latter has great potential for the production of activated carbons implemented in the adsorption of polluting species in aqueous solutions.^24–26^

Kinetic and thermodynamic studies and interaction mechanisms associated with pesticide adsorption processes using activated carbon, have been widely addressed.^27–29^ Despite this, no studies have been found that report kinetic or thermodynamic data regarding the adsorption process of the pesticide Lufenuron 50-EC using activated carbon. Consequently, no studies related to the interaction mechanism between activated carbon and Lufenuron have been found. Due to this, in this work, the adsorption kinetics and thermodynamic parameters of the ACs synthesized and used in the adsorption of the pesticide Lufenuron in a previous study^30^ were determined. In addition, a physical-chemical interaction mechanism between the ACs and the pesticide Lufenuron is proposed, confirming what had been concluded about the potential capacity of these ACs to adsorb contaminants in aqueous solutions. In this previous study,^30^ the precursor was rendered by drying, quartering, grinding, and sieving processes to obtain the desired particle size (0.85–2 mm). The activation process significantly influences the properties of activated carbons (ACs). Physically activated carbons are affected by activation temperature, while chemically activated carbons depend on residence time and the impregnation ratio for BET surface area development. Oil palm shell is identified as a suitable precursor for AC production due to its high fixed carbon content and low ash. While KOH activation reduced the BET surface area by widening pores, it introduced functional groups such as carboxylic (−COOH), enhancing adsorption capacity. High adsorption levels (94–96.9%) were achieved due to strong interactions between functional groups and Lufenuron. Adsorption efficiency depends not only on the surface area but also on the nature of the functional groups.

Previous literature^31,32^ has demonstrated that regeneration efficiency can vary significantly depending on the regeneration method (e.g., thermal, chemical, or biological) and the type of adsorbate involved. For instance, thermal regeneration often leads to a partial loss of adsorption capacity due to structural changes in the adsorbent, while chemical methods might enhance regeneration for specific adsorbates. Exploring these aspects for Lufenuron adsorption would provide a more comprehensive understanding of the practical viability of these ACs.

## 2 Experiments

### 2.1 Materials and Characterization

The activated carbons utilized in this study were prepared and characterized as detailed in a previous study,^30^ which also examined the Lufenuron adsorption process. The operating parameters for obtaining these activated carbons and the activation yields are presented in Table 1.^30^ These parameters were chosen considering previous research related to the production of activated carbon from oil palm shell.^33,34^ The samples named AC-800-2, AC-900-2, AC-750-1.5-3:1, and AC-800-1-2:1 were used. The adsorption experiment^30^ involved mixing different amounts of activated carbon (0.05 to 0.45 g) with a 50 mL solution of Lufenuron (10 ppm) in distilled water. The mixtures were placed in 50 mL glass containers and agitated in a shaker at 170 rpm for 20 h at room temperature. This setup aimed to evaluate the adsorption capacity of activated carbon under controlled conditions. The methodology involved characterizing precursors and activated carbon samples using ASTM standards to determine proximate and ultimate properties, including volatile matter, fixed carbon, ash, HHV, and elemental composition. Functional groups were analyzed by FTIR using a Jasco FT/IR-4100; textural properties (SBET, VT, SP) were analyzed by nitrogen adsorption at 77 K using Micromeritics 3Flex; and crystalline structure was analyzed by XRD with Rigaku Ultima IV equipment. Raman spectroscopy with an InVia Raman Microscope assessed graphitization and structural defects, while SEM using a JEOL JSM-6490LV examined surface morphology. This comprehensive approach ensured a detailed understanding of the adsorption capabilities of the materials. This information can be found in greater detail in our recently published study.^30^

**Table 1:** Activation Mass Yields, Dry Base, db

Process activation	Sample	Temperature (°C)	Residence time (h)	Impregnation rate (KOH/Biochar)	AC yield (% w/w)
Physical	AC-800-2	800	2.0	N/A	24.9
	AC-900-2	900	2.0	N/A	20.2
Chemical	AC-750-1.5-3:1	750	1.5	3:1	22.1
	AC-800-1-2:1	800	1.0	2:1	25.9

### 2.2 Adsorption Kinetics

Adsorption kinetics is considered to be one of the main properties that control the rate of adsorption and the adsorption effectiveness of solutes. The pseudo-first-order, pseudo-second-order, and Elovich models are the most used. The intraparticle diffusion model applies when adsorption is limited by mass transfer.^35,36^ For the adsorption kinetic procedure, aliquots were taken at specific time intervals, and the concentrations of Lufenuron were measured. The amount of adsorption at time *t*, *q*_*t*_ (mg/g), was calculated by eq 1.

3 $$q_{t}=\frac{\left(C_{0}-C_{t}\right)V}{m}$$

Where *C*_0_ and *C*_*t*_ (mg/L) are the concentrations of the pesticide at the initial time and at any time *t,* respectively, *V* is the solution volume, and *m* is the AC mass used.

In this work, the adsorption kinetics of Lufenuron in AC was analyzed according to the pseudo-first-order and pseudo-second-order models (eqs 2 and 3 respectively), which are represented in Table 2,^37,38^ where *k*_1st_ and *k*_2nd_ (g/(mg min)) are the pseudo-first- and pseudo-second-order rate constants, respectively, *q*_*e*_ (mg/g) and *q*_*t*_ (mg/g) are the values of the amount adsorbed per unit mass at equilibrium and at any time *t*, respectively. Both models are empirical and designed for simplicity, making them effective at describing certain systems but inadequate at capturing the complexities introduced by pore diffusion resistances. In real-world applications, particularly for microporous and mesoporous materials, more advanced models, such as those implemented in previous research^39,40^ are often required to account for these effects comprehensively.

**Table 2:** Kinetic Models

Models	Nonlineal form	Linear form	Equation
Pseudo-first order	$\frac{dq_{t}}{dt}=k_{1st}\left(q_{e}-q_{t}\right)$	1 $\ln\left(q_{e}-q_{t}\right)=\ln q_{e}-k_{1st}t$	(1)
Pseudo-second order	$\frac{dq_{t}}{dt}=k_{2nd}{(q_{e}-q_{t})}^{2}$	2 ${\frac{t}{q}}_{t}=\frac{1}{k_{2nd}{q_{e}}^{2}}+\frac{1}{q_{e}}t$	(2)

### 2.3 Thermodynamic Parameters

The thermodynamic analysis of adsorption allowed us to estimate if the adsorption process is spontaneous or nonspontaneous and the influence of temperature (298, 313, and 323 K) on it. To achieve this, the change in the Gibbs standard free energy was determined by following basic equations:

4 $$\Delta G^{{}^{\circ}}=\Delta H^{{}^{\circ}}-T\Delta S^{{}^{\circ}}$$

5 $$\Delta G^{{}^{\circ}}=-RT\;\ln K^{0}$$

By equating eqs 4 and 5, it is obtained:

6 $$-RT\;\ln K^{0}=\Delta H^{{}^{\circ}}-T\Delta S^{{}^{\circ}}$$

Solving for *K* ^0^, it is obtained from the Van’t Hoff eq 7:

7 $$\ln K^{0}=\frac{\Delta S^{{}^{\circ}}}{R}-\frac{\Delta H^{{}^{\circ}}}{RT}$$

8 $$K_{d}=\frac{C_{a}}{C_{e}}$$

Where Δ*G*° represents the standard Gibbs free energy, Δ*H*° is the standard enthalpy, Δ*S°* is the standard entropy, *T* is the temperature in Kelvin, *K*_*d*_ (L/kg) is the equilibrium distribution coefficient,^41^ defined as the ratio of the amount of adsorbate in the adsorbent to that in the solution. *R* is the gas constant (8.314 J/mol K), *C*_*a*_ is the adsorbate adsorbed per unit mass of the adsorbent, and *C*_*e*_ is the equilibrium adsorbate concentration in the aqueous phase.

To perform the thermodynamic analysis, the dimensionless equilibrium constant (*K* ^0^) was obtained from *K*_*d*_ using appropriate standard states as shown in eq 9:

9 $$K^{0}=K_{d}\frac{\gamma_{a}}{\gamma_{e}}\left(\frac{C_{aq}^{0}}{C_{ad}^{0}}\right)$$

Where *γ_a_* and *γ_e_* are the activity coefficients (assumed to be 1 for dilute solutions), $C_{aq}^{0}$ is the standard state for the aqueous phase (1 mol/L), and $C_{ad}^{0}$ is the standard state for the adsorbed phase (1 mol/kg). This conversion ensures that *K* ^0^ is dimensionless, making its use valid in thermodynamic equations.^42,43^

Under these considerations, the relationship is simplified as follows:

10 $$K^{0}=K_{d}$$

It is important to note that, numerically, *K* ^0^ is equal to *K*_*d*_ when the latter is expressed in L/kg, but *K* ^0^ is dimensionless.

Carrying out the adsorption at three different temperatures is enough to make an approximate interpretation of the thermodynamic behavior of the process.^44,45^ The initial concentration of Lufenuron in the solution was set at 10 ppm, after the adsorption process, the final concentrations of the pesticide were measured to determine the amounts of pesticide removed. Then, the Δ*H*° and Δ*S*° values were obtained from the slope and intercept of the plot of ln *K* ^o^vs 1/*T.*

### 2.4 Physical-Chemical Interaction Mechanism

The interaction mechanism was carried out, considering the nature of the activation, the BET surface area, and the surface functional groups of the ACs and the Lufenuron molecule, as established in a previous study.

## 3 Results and Discussion

### 3.1 Characterization

The characterization of the samples was conducted in a previous study^30^ and a summary of these results is presented below.

With the proximate and ultimate analysis, it was found that the ACs have a good fixed carbon content and low ash content—characteristics that favor the generation of surface area and porosity, which, in turn, favors the adsorption capacity of the ACs. Regarding the FTIR, it was found that the functional groups associated with the samples are —OH due to the presence of water in hydration at 105 °C, carboxyl groups (—COOH), carbonyl (C=O), and (C—O).

BET surface area, pore volume, and pore size distribution were determined by N_2_ adsorption–desorption isotherms at 77 K.^46^ As can be seen in Table 3, this study reports exceptionally high adsorption capacities (1011–1352 mg/g) compared to other studies (41–48 mg/g). Despite the stark contrast, the adsorption capacities in other works are significant within their specific contexts, as they likely reflect optimized adsorbent–adsorbate compatibility and specific experimental conditions. For example, the TSAC sample, with *q_e_* = 41 mg/g, may have been tailored for a different adsorbate or experimental setup, making its performance noteworthy in that domain. Parallel to this, a higher *S*_BET_ often correlates with greater adsorption due to the increased available surface sites. However, this relationship is not linear: CC-FD (1209 m^2^/g) has a high surface area but a modest q_e_ = 48 mg/g, indicating other limiting factors, such as adsorbate–adsorbent affinity or pore size distribution. In contrast, AC-750-1.5-3:1 (22 m^2^/g) achieves *q*_*e*_ = 1167 mg/g suggesting that chemical interactions or functional groups dominate adsorption over the physical surface area.

**Table 3:** Comparison of BET Analysis and Adsorption Capacity (*q*_*e*_) of Oil Palm Shell AC with Other Adsorbents

Sample	*S*_BET_ (m^2^/g)	*V*_T_ (cm^3^/g)	*S*_P_ (nm)	*q*_*e*_ (mg/g)	Source
TSAC	660	0.620	1.410	41	^27^
CC-FD	1209	0.519	<2	48	^33^
AC-750-1.5-3:1	22	0.010	7.336	1167	This study
AC-800-2	421	0.027	3.753	1011	
AC-800-1-2:1	90	0.027	6.498	1149	
AC-900-2	548	0.080	5.420	1352	

X-ray diffraction (XRD) analysis showed peaks at 2θ of 20°–30° and 40°–45°, corresponding to diffraction peaks of the graphitic and disordered graphitic plane. This behavior is representative of an amorphous carbon crystalline structure, found properly in activated carbon.^47^

Raman spectroscopy analysis showed two characteristic peaks at 1350 cm^–1^ and 1560 cm^–1^ which are called the D and G bands, respectively; the G band is indicative of a large presence of O—H functional groups on the activated carbon surface. These peaks are mainly attributed to the presence of sp^2^ carbon–carbon bonds in disordered microcrystalline domains of AC.^48^ As the pyrolysis temperature increases, the I_D_/I_G_ ratio increases from 0.89 to 0.94. These results imply a higher degree of defects and disorder in the atomic arrangement of the activated carbons; at the same time, they imply a lower degree of graphitization as the activation temperature increases. This occurs because more carbon could be gasified, giving rise to more defects within the carbon plane.^49^

SEM analysis showed an abundant presence of holes of different sizes, as well as cracks and crevices, on the surface of the samples obtained by chemical activation (AC-750-1.5-3:1 and AC-800-1-2:1) in comparison with activated carbons produced through the physical activation process (AC-800-2 and AC-900-2). This observation is attributed to the different activation processes, especially the use of KOH as an impregnation agent in the chemical activation process.

Considering previous research,^50,51^ the relevance of the zero charge point (pH_pzc_) analysis in pesticide adsorption processes using activated carbon has been demonstrated due to its influence on the pH of the solution and the electrostatic interactions between the adsorbent and the contaminants. The pH_pzc_ determines the pH range in which the activated carbon surface is positively or negatively charged, which directly affects the affinity of the material toward anionic or cationic species present in the solution. Although this analysis was not performed in the present study, its consideration could provide additional information on the adsorption mechanisms and the optimal conditions to maximize pesticide removal efficiency.

### 3.2 Adsorption Kinetics

**T**he experimental data on the adsorption of Lufenuron at room temperature using the selected activated carbons AC-800-2, AC-900-2, AC-750-1.5-3:1, and AC-800-1-2:1 obtained physically and chemically, are presented in Figures 1 and 2. In these figures, the adjusted pseudo-first-order (PFO) and pseudo-second-order (PSO) models were graphed to compare them with the experimental values.

**Figure 1:**
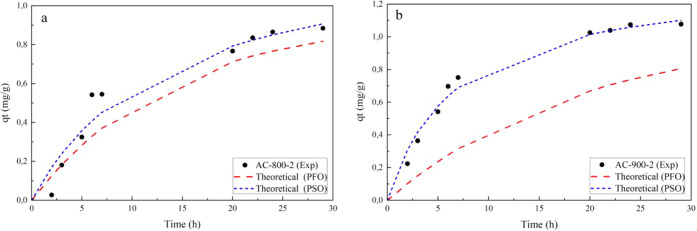
Adsorption kinetics of Lufenuron from physically activated carbon: (a) AC-800-2 and (b) AC-900-2.

**Figure 2:**
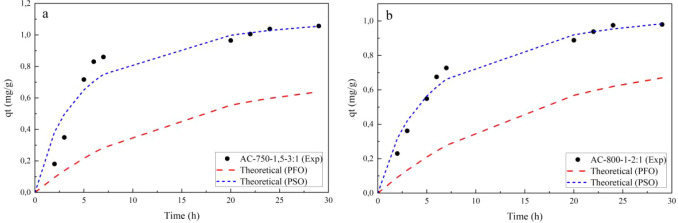
Adsorption kinetics of Lufenuron from chemically activated carbon: (a) AC-750-1.5-3:1 and (b) AC-800-1-2:1.

As seen in Figures 1 and 2, it was found that, for the four ACs, the amount of Lufenuron pesticide adsorbed achieved equilibrium in less than 7 h, regardless of the method used for activation. In the adsorption of Lufenuron, the activated carbons AC-900-2, AC-750-1.5-3:1, and AC-800-1-2:1 present a similar kinetic behavior, starting with a high adsorption of the pesticide at relatively low times and reaching equilibrium with an amount of Lufenuron adsorbed. Additionally, a better fit was observed using the pseudo-second-order kinetic model for these ACs. On the other hand, the activated carbon AC-800-2, although it presented a kinetic behavior similar to that of the other ACs in the adsorption of Lufenuron, showed a similar fit of the experimental data for both PFO and PSO kinetic models.

Additionally, in Figures 1 and 2, it is observed that, for the activated carbons AC-900-2 and AC-750–1.5-3:1, the Lufenuron adsorption process presented higher adsorption values, 1.077 and 1.057 mg/g, respectively. In contrast, the activated carbons AC-800-2 and AC-800-1-2:1 presented lower Lufenuron adsorption values, 0.084 and 0.980 mg/g, respectively. The different characteristics of these activated carbons influence the different adsorption behaviors of the Lufenuron pesticide.

Pseudo-first-order and pseudo-second-order models were utilized to analyze the kinetic behavior of the Lufenuron adsorption process onto activated carbons produced in this study. The adsorption capacity *q*_*e*_, adsorption rate *k*_1st_ and *k*_2nd_ constants of these kinetic models were determined together with the correlation factors *R* ^*2*^ and are found in Table 4.

**Table 4:** Pseudo-First-Order and Pseudo-Second-Order Model Equation Constants and Correlation Coefficients for Lufenuron Adsorption

	PFO model			PSO model		
Sample	*k*_1st_	*q*_*e*_ (mg/g)	*R* ^2^	*k*_2nd_	*q*_*e*_ (mg/g)	*R* ^2^
AC-800-2	0.072	0.929	0.960	0.054	1.334	0.910
AC-900-2	0.052	1.032	0.939	0.108	1.358	0.982
AC-750-1.5-3:1	0.069	0.736	0.873	0.190	1.214	0.931
AC-800-1-2:1	0.059	0.815	0.936	0.161	1.165	0.986

The values of *q*_*e*_ determined with the pseudo-second-order kinetic model fit better to the experimental *q*_*e*_ than the values determined with the pseudo-first-order model. The trend lines of the pseudo-second-order plots, *t/q*_*t*_ vs t, revealed *R* ^2^ values between 0.91 and 0.99, while for the pseudo-first-order model, ln­(*q*_*e*_ *– q*_*t*_) vs *t*, *R* ^2^ values were obtained between 0.88 and 0.96, which are lower than those obtained with the pseudo-second-order model. These values can be seen in Table 4 and Figure 3.

**Figure 3:**
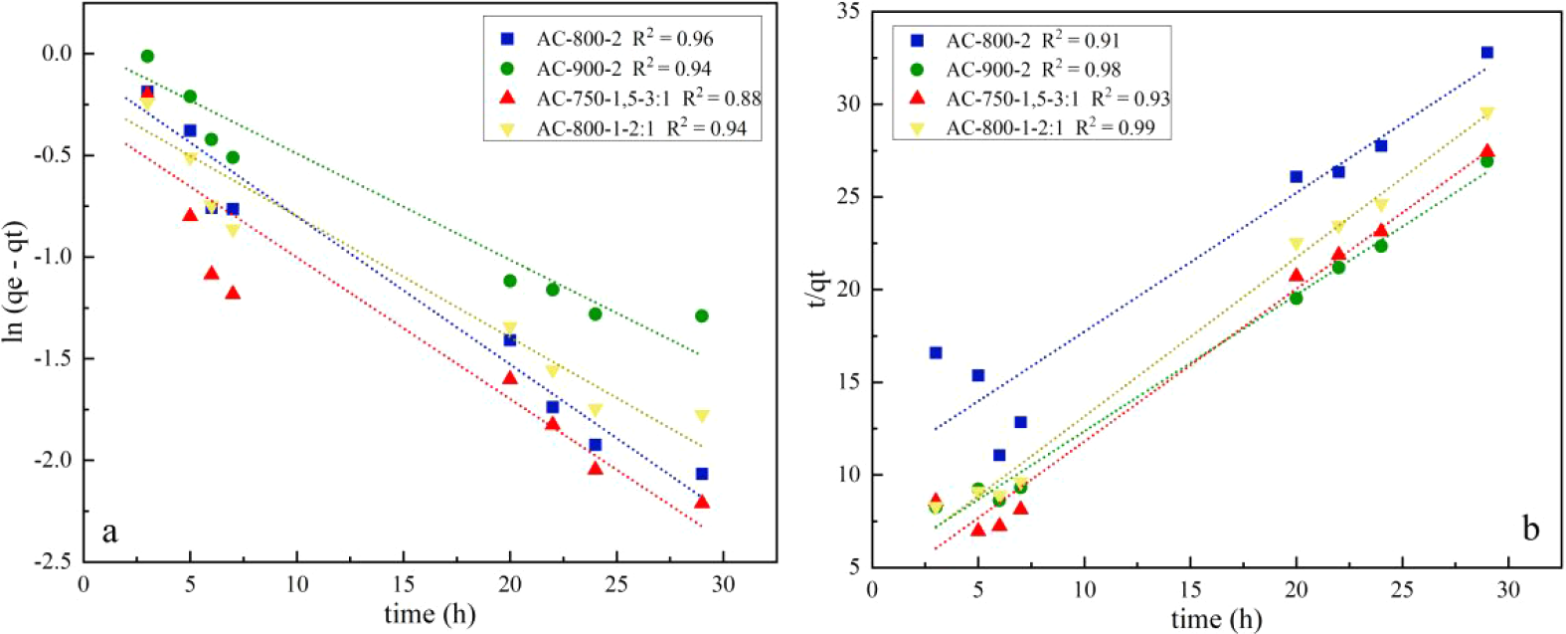
Adsorption kinetics of Lufenuron onto activated carbons (room temperature): (a) pseudo-first order and (b) pseudo-second order.

The experimental data are best represented overall by the pseudo-second-order kinetic model; this assumes that the Lufenuron adsorption process onto activated carbons produced in this work is related to the chemisorption process. Chemisorption is a process in which atoms or molecules of one substance adhere to the surface of another substance due to a chemical reaction between them. This suggests that Lufenuron must have a chemical affinity for the surface of activated carbons, which means that the Lufenuron atoms or molecules react with the chemical groups on the AC surface and form strong chemical bonds, such as covalent bonds, and this is comparable to the results of previous investigations.^18,52,53^

Although the correlation coefficients (*R* ^2^) for the pseudo-first-order and pseudo-second-order kinetic models are really close, further statistical analysis is needed to confirm the most suitable model for the adsorption process. To address this, the sum of squares (SS) and chi-square techniques were implemented based on previous investigations;^54,55^ the results of these techniques are given in Table 5.

**Table 5:** Statistical Metrics for Pseudo-First-Order and Pseudo-Second-Order Models in Lufenuron Adsorption

	PFO model		PSO model	
Sample	Sum of Square	Chi-Square	Sum of Square	Chi-Square
AC-800-2	0.113	0.339	0.053	0.204
AC-900-2	0.941	2.660	0.019	0.043
AC-750-1.5-3:1	1.689	5.268	0.096	0.197
AC-800-1-2:1	1.013	3.350	0.020	0.046

As observed in Table 5, PSO shows lower SS and chi-square values compared to PFO, indicating that the PSO model fits the experimental data better in all samples, especially in samples AC-900-2, AC-750-1.5-3:1, and AC-800-1-2:1, where the sum of squares and chi-square values are lower. These results could support the choice of the PSO model as the most suitable one to describe the adsorption kinetics of the experimental data in this work.

A previous study^56^ on the photodegradation of Lufenuron showed a degradation rate of 79.8% in just 2 h, with a kinetic constant (*k*) of 0.66 h^–1^. This indicates that photodegradation is a rapid process, with a half-life (*t*_1/2_) of 1.05 h, demonstrating high efficiency in a short time, making it suitable for applications where rapid removal is required. In contrast, according to the PSO model implemented in this study, the kinetic constants (*k*_2_) vary between 0.054 and 0.190 g mg^–1^ h^–1^, depending on the type of AC. Although the adsorption is slower, the high *q_e_* values (1.165–1.358 mg/g) suggest a high retention capacity of Lufenuron, offering a sustainable solution for the removal and retention of Lufenuron, with high adsorption capacity.

### 3.3 Thermodynamic Parameters

Thermodynamic parameters play a critical role in determining the feasibility and nature of the adsorption process. The Gibbs free energy (Δ*G*°) reflects the degree of spontaneity of the process. Negative values of Δ*G*° indicate that the process is spontaneous and therefore favors adsorption, while positive values suggest that the process is not spontaneous and consequently, adsorption is disfavored.

The enthalpy (Δ*H*°) is related to the thermal nature of the reaction. Negative values of Δ*H*° indicate that the process is exothermic, while positive values indicate that the reaction is endothermic.

On the other hand, the entropy (Δ*S*°) allows estimating the degree of changes in the surface of the adsorbent. Positive values reflect the irreversibility of the process, while negative values suggest a greater probability of the reversibility of the process.

The three thermodynamic parameters that characterize the adsorption process at the three temperatures investigated for each of the selected activated carbons are presented in Table 6.

**Table 6:** Thermodynamic Parameters

Sample	*T* (K)	Δ*G*° (kJ/mol)	Δ*H*° (kJ/mol)	Δ*S*° (J/mol K)
AC-800-2	298	1.04	14.63	45.58
	313	0.36		
	323	–0.09		
AC-900-2	298	–2.95	–47.45	–149.25
	313	–0.71		
	323	0.77		
AC-750-1.5-3:1	298	–1.29	–18.65	–58.20
	313	–0.42		
	323	0.15		
AC-800-1-2:1	298	–1.67	–21.30	–65.82
	313	–0.68		
	323	–0.02		

For sample AC-800-2, the increase in temperature favored the adsorption process. This effect is due to the fact that, at higher temperatures, a negative value of Δ*G*° was obtained at 323 K, which means that there is spontaneous adsorption in the process. Similar results were found in refs. 41 and 44. Also, negative values of Δ*G*° between 0 and −20 kJ/mol represent electrostatic interactions between the active sites of the adsorbent and the adsorbed compound, that is, it presents a physisorption.^57^ Therefore, the adsorption between activated carbon AC-800-2 and the pesticide at the temperature of 323 K was carried out through a spontaneous and physical process governed mainly by electrostatic forces.

In contrast, for samples AC-900-2, AC-750-1.5-3:1, and AC-800-1-2:1, the increase in temperature did not favor the adsorption of the pesticide Lufenuron, because positive results were obtained for Δ*G*°, revealing that the process is not spontaneous and that some external force is required to promote the adsorption of this adsorbate.^58^

The variation in enthalpy and entropy during the adsorption of the pesticide Lufenuron shows significant differences between the samples. In the case of activated carbon AC-800-2, the positive values of Δ*H*° and Δ*S*° show that the adsorption process of Lufenuron is endothermic and irreversible. Similar results were presented by Nguyen.^52^ This suggests that the solid–liquid interface is in a state of increased disorder. In contrast, the activated carbons AC-900-2, AC-750-1.5-3:1, and AC-800-1-2:1 presented negative values of Δ*H*° and Δ*S*°, suggesting that the adsorption of Lufenuron is exothermic and presents a greater probability of reversibility. Negative values indicate that no significant changes occur in the internal structure of the adsorbent and that desorption could occur. This implies that some molecules adsorbed in the solid phase may return to the liquid phase, resulting in a decrease in the amount of the adsorbed adsorbate. These negative values mean that van der Waals forces and hydrogen bonds played an important role in this adsorption process.^27^

### 3.4 Physical-Chemical Interaction Mechanism

The data^30^ obtained from the FTIR showed some details about the surface interactions in activated carbon produced by physical activation and chemical activation.

#### 3.4.1 Mechanism for the Adsorption of Lufenuron with Physically Activated Carbons

The proposed mechanism for the adsorption of the pesticide Lufenuron with physically activated carbons is presented in Figure 4.

**Figure 4:**
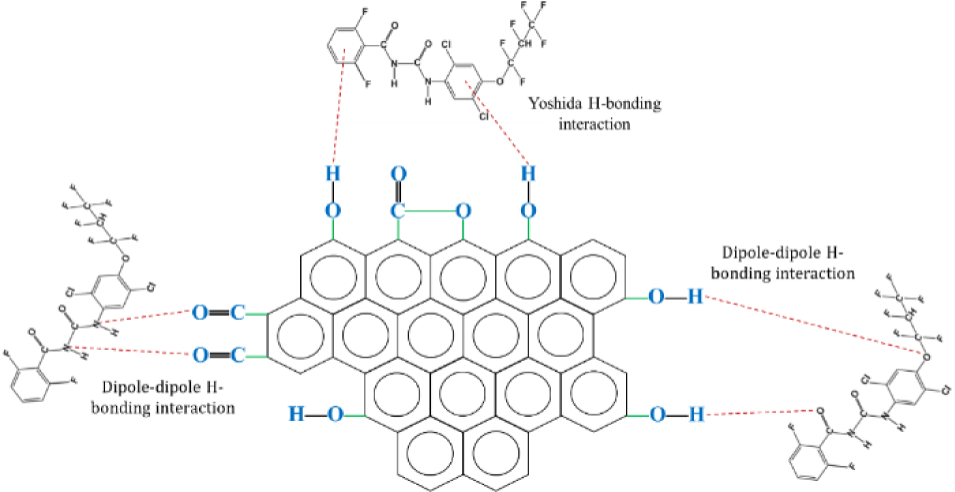
AC-Lufenuron physicochemical interaction mechanism using physical activation.

In both activation processes, the prominent bands of hydroxyl functional groups (—OH) present on the surface, which appear around 3424 cm^–1^ in the IR spectrum,^30^ suggest the possible formation of hydrogen bonds with the parts of Lufenuron that contain atoms of oxygen and/or nitrogen in its structure (dipole–dipole H-bond). Furthermore, it is possible that these hydroxyl groups on activated carbon interact with the aromatic rings of Lufenuron, which is known as Yoshida H-bonding.^59^

Additionally, carbonyl functional groups (C=O) are identified between the bands 1562 and 1637 cm^–1^ in the IR spectrum.^30^ These carbonyl groups are polar and can establish weak interactions with other molecules through dipole–dipole forces. In the case of Lufenuron, the C=O group can interact with other molecules that have hydrogen atoms or groups with oxygen and/or nitrogen atoms, which are capable of forming hydrogen bonds or dipole–dipole forces.

#### 3.4.2 Mechanism for the Adsorption of Lufenuron with Chemically Activated Carbons

The adsorption mechanism of Lufenuron on chemically activated carbons, as shown in Figure 5 presents similarities to the mechanism observed in activated carbons produced by physical activation.

**Figure 5:**
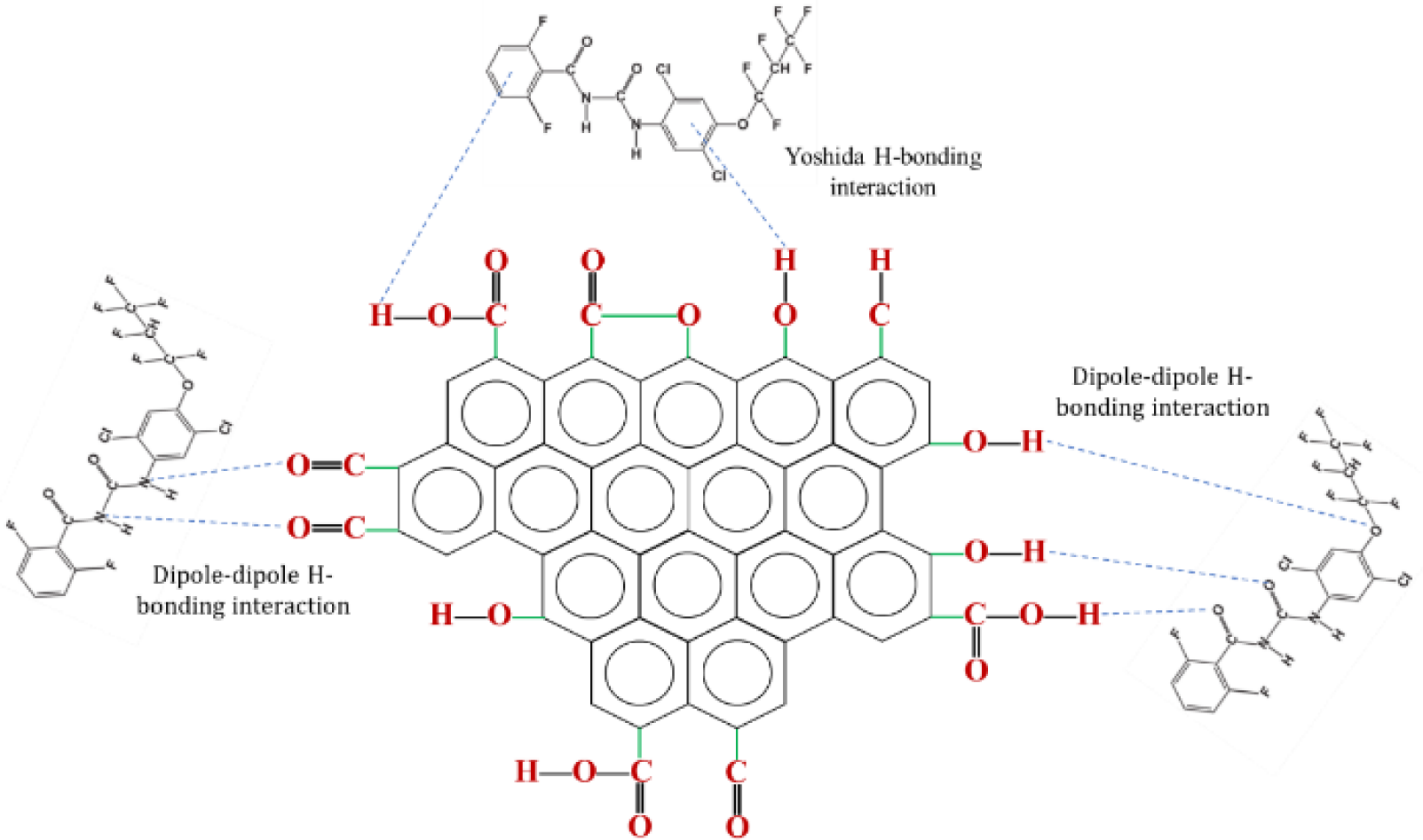
AC-Lufenuron physicochemical interaction mechanism using chemical activation.

Both types of activated carbons exhibit interactions with hydroxyl (−OH) functional groups present on their surfaces, resulting in the formation of dipole–dipole hydrogen bonds. Furthermore, it is highlighted that the carbonyl functional groups (C=O) are more prominent in the activated carbons impregnated with KOH (AC-750-1.5-3:1 and AC-800-1-2:1) compared with the samples physically activated (AC-800-2 and AC-900-2). This suggests greater interaction through dipole–dipole forces in the chemically activated samples, which could influence the differences in the adsorption processes between both categories of activated carbons. The use of KOH as an activating agent to produce activated carbon^60,61^ has proven to be more effective in the development of large surface areas, compared to other agents such as FeCl_3_ and H_3_PO_4_, enhancing the H-bonding interactions between adsorbate and adsorbent. In addition, these activated carbons have demonstrated adsorption processes with better experimental fit to the pseudo-second-order kinetic model, suggesting a large contribution of chemical adsorption in the process. This is in line with the results obtained in this work.

Regarding the carboxyl groups (—COOH) present in the samples obtained by chemical activation, bands are observed around 2950 cm^–1^ in the IR spectrum,^30^ which suggests that these groups could be establishing hydrogen bonds with the functional groups of Lufenuron that contain atoms of oxygen and/or nitrogen (dipole–dipole H-bond). Similar to the hydroxyl groups, the carboxyl groups could be interacting with the positive charges on Lufenuron, leading to ionic and dipole–dipole interactions.

The adsorption process of the pesticide Lufenuron 50-EC on the surface of activated carbons is due to a series of previously mentioned molecular interactions. It is important to highlight that the functional groups present in Lufenuron can be arranged in a way that facilitates its interaction with the functional groups of activated carbon, which leads to the formation of hydrogen bonds and other intermolecular interactions. Although activated carbons produced by physical activation exhibit considerably higher BET surface areas (421 and 548 m^2^/g) compared to chemically activated carbons (22 and 90 m^2^/g), other factors, such as the nature of activation and the specific functional groups, may be playing a fundamental role in the observed adsorption capacities and performances.

In the case of activated carbons produced by physical activation, the high surface area represents a greater number of active sites available for the adsorption of Lufenuron. This characteristic, together with the presence of hydroxyl (—OH) and carbonyl (C=O) functional groups on the surface of these carbons, could promote strong interactions with Lufenuron. The abundance of available active sites and the polar nature of these functional groups could contribute to a strong interaction between the activated carbon and the pesticide, which significantly increases its adsorption capacity.

On the other hand, for activated carbons produced by chemical activation, despite having smaller surface areas, the presence of specific functional groups, such as carboxyl groups (—COOH), generated through the use of KOH in the impregnation process, seems to allow an effective interaction with Lufenuron. These carboxyl groups can establish hydrogen bonds and other interactions with the pesticide, which increase its adsorption capacity despite having a smaller surface area.

## 4 Conclusions

The activated carbons successfully prepared from oil palm shells in the first part of this study can be used as promising adsorbents for the removal of Lufenuron from aqueous solutions. The adsorption process of Lufenuron followed the Langmuir isotherm model and the pseudo-second-order kinetics, indicating that it was monolayer adsorption and mainly controlled by chemisorption. In addition, this adsorption process was spontaneous, exothermic, and had a high probability of reversibility for samples AC-900-2, AC-750-1.5-3:1, and AC-800-1-2:1, with van der Waals forces and hydrogen bonds playing a major role in the interaction of Lufenuron-AC. The opposite was the case for sample AC-800-2 where the adsorption process required an increase in temperature to be spontaneous; in this case, the process was endothermic and irreversible. The high percentages of adsorption observed in both ACs could be explained by the strong interactions of the superficial functional groups with Lufenuron. In the case of physically activated carbon, the high surface area provides more sites available for these interactions, and in the case of chemically activated carbon, although the surface area is lower, the functional groups introduced using KOH improve the adsorption capacity of these activated carbons.

## Supplementary Material



## Notes

Adsorption isotherms of the samples investigated in this work, Langmuir and Freundlich constants for the adsorption of Lufenuron, and Van’t Hoff plots used for the thermodynamic evaluation (PDF)
