# Supplementary material for: Oil Palm Shell-Derived Activated Carbon: Adsorption Kinetics, Thermodynamics, and Interaction Mechanism for Lufenuron 50-EC Pesticide
Source: ACS Omega. 2026 Jan 27;11(5):7005–13. doi: 10.1021/acsomega.4c10096 (PMC12902967; doi:10.1021/acsomega.4c10096)
Supplement: Supplementary file 1 [file ao4c10096_si_001.pdf]

Supporting Information

Oil Palm Shell-Derived activated carbon: Adsorption kinetics, thermodynamics and interaction mechanism for Lufenuron 50-EC pesticide

David Nuñez-Vargas, Juan Barraza-Burgos*, Juan Guerrero-Perez, Luis Diaz, Ajay K. Dalai and Venu Babu Borugadda

ACS Omega

The following information on adsorption isotherms and Langmuir constants was taken from our recently published paper "Adsorption of Lufenuron 50-EC Pesticide from Aqueous Solution Using Oil Palm Shell-Derived Activated Carbon" (https://doi.org/10.3390/ma17215389):

Adsorption Isotherms for Selected AC Obtained by Physical Activation

Figure S1 shows the experimental equilibrium adsorption isotherms of Lufenuron pesticide for activated carbons AC-800-2 and AC-900-2, which are compared with the Langmuir and Freundlich mathematical models.

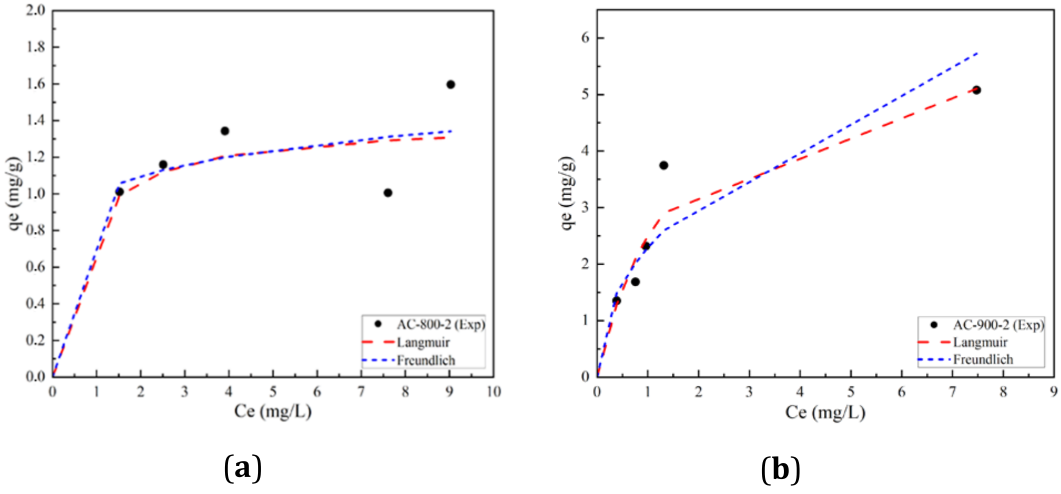

Figure S1. Adsorption isotherms of Lufenuron using physical AC (**a**) AC-800-2 and (**b**) AC-900-2.

Adsorption Isotherms for Selected AC Obtained by Chemical Activation

Figure S2 shows the experimental equilibrium adsorption isotherms of Lufenuron pesticide for activated carbons AC-750-1.5-3:1 and AC-800-1-2:1, which are compared with the Langmuir and Freundlich mathematical models.

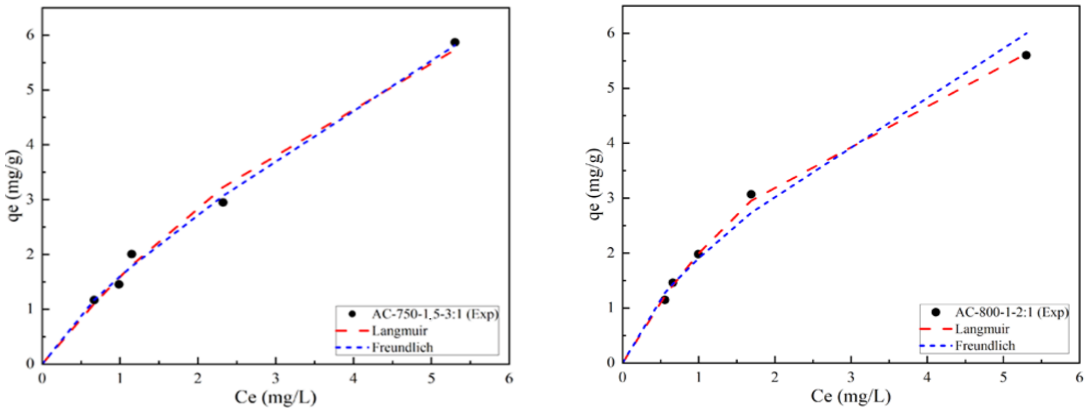

(**a**) (**b**)

Figure S2. Adsorption isotherms of Lufenuron using chemical AC (**a**) AC-750-1.5-3:1 and (**b**) AC-800-1-2:1.

Langmuir and Freundlich constants for the adsorption of Lufenuron.

Table S1 presents the Langmuir and Freundlich isotherm constants obtained from the adsorption equilibrium data of Lufenuron onto the prepared activated carbon. These parameters describe the adsorption capacity and affinity of the adsorbent surface under the studied conditions.

Table S1. Langmuir and Freundlich constants for the adsorption of Lufenuron.

Model	Parameter	Sample			
		AC-800-2	AC-900-2	AC-750-1.5-3:1	AC-800-1-2:1
Langmuir	q_m	1.40	6.09	14.54	9.77
	K_L	1.61	0.69	0.12	0.26
	R^2	0.82	0.98	0.84	0.99
	R_L	0.06	0.10	0.42	0.26
Freundlich	K_F	1.00	2.29	1.60	1.91
	$1/n_F$	0.13	0.46	0.77	0.69
	R^2	0.25	0.84	0.98	0.98

Van't Hoff Plots for the Thermodynamic Evaluation.

Figure S3 shows the Van't Hoff plots corresponding to the adsorption of Lufenuron at different temperatures. These graphs were used to calculate the thermodynamic parameters (ΔH°, ΔS°, and ΔG°), providing insight into the nature and spontaneity of the adsorption process.

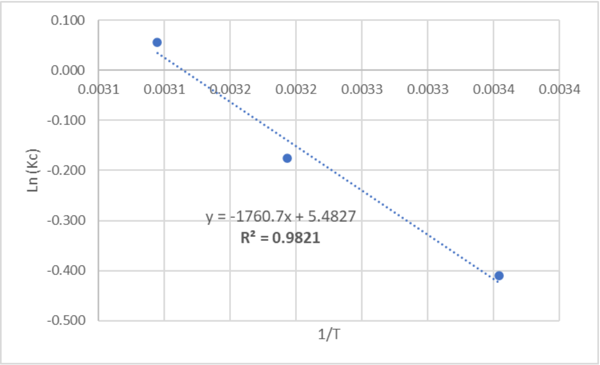

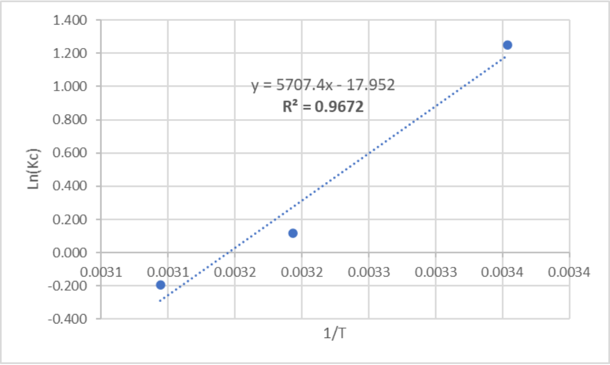

(**a**) (**b**)

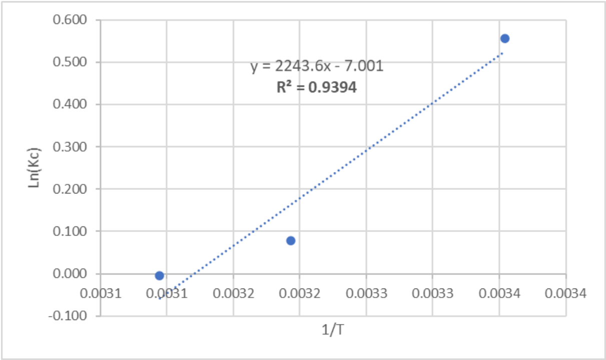

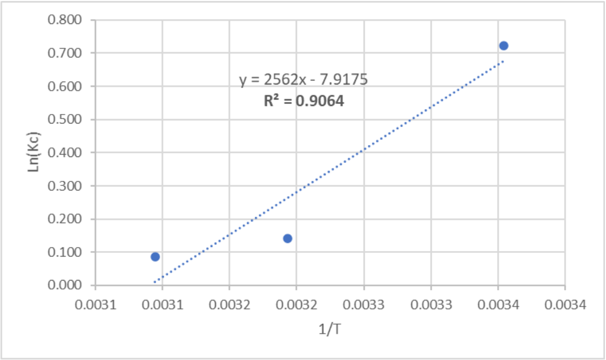

(**c**) (**d**)

Figure S3. Van't Hoff plots for Physical AC (**a**) AC-800-2 and (**b**) AC-900-2 and Chemical AC (**c**) AC-750-1.5-3:1 and (**d**) AC-800-1-2:1.

We confirm that the standard state was considered at 298 K for calculating the thermodynamic parameters. However, to evaluate the temperature dependence, additional calculations were also conducted at 313 K and 323 K.
